# Closing the circle: a key collaborative opportunity for general practice and psychiatry

**DOI:** 10.1192/bjb.2021.116

**Published:** 2022-12

**Authors:** André Tylee, Alan Cohen, Lydia Thurston

**Affiliations:** 1 King's College London, UK; 2 Oxfordshire Mind, Oxford, UK; 3 Oxford Health NHS Foundation Trust, UK

**Keywords:** Primary care, history of psychiatry, multiprofessional training, depressive disorders, anxiety disorders

## Abstract

Two of the authors were general practitioners (GPs) in the 1980s, when there was much interest in consultation, stimulated by the psychoanalyst Michael Balint. Around one in five psychiatrists worked in consultation liaison in general practice at that time, but in the 1990s this was stopped to increase the focus on psychosis. However, the Royal College of Psychiatrists and Royal College of General Practitioners have a strong history of collaboration, and many psychiatrists, nurses and GPs trained together in the national Trailblazers programme, focusing on service delivery in all areas of mental health. Recent proposals for mental health community collaborative networks from the NHS provide an opportunity for psychiatrists to work with GPs and a range of other professionals once more, for complex non-psychotic illness that cannot be helped by Improving Access to Psychological Therapies services. The circle is closing for GPs like us, who were working in the 1980s.

Recent proposals for mental health community collaborative networks^1^ provide an opportunity for psychiatrists to work with general practitioners (GPs) once more for serious mental illness, both psychotic and non-psychotic. The circle is closing for GPs like A.T. and A.C., who were working in the 1980s.

Fifty years ago, psychoanalyst Michael Balint helped GPs examine their consultation style to understand doctor–patient relationships.^2^ Understanding the context and nuances of the consultation were core to primary care. Then, 20% of psychiatrists worked in liaison attachment to general practice, sometimes running Balint-style groups.^3^

A 1989 White Paper^4^ introduced the purchaser provider split, establishing GP fundholding, which allowed larger practices to purchase counsellors and other psychological support such as community psychiatric nurses (CPNs). In the 1990s, a CPN report^5^ identified a need for better psychosis care, resulting in psychiatric teams and CPNs being pulled from general practice to work in early psychosis intervention and crisis support teams.

Despite this, at the turn of the century, hundreds of GPs, psychiatrists, CPNs, practice nurses and other healthcare professionals in England still underwent joint training in the Trailblazers programme,^6^ to develop better collaboration between mental health services and primary care. The Royal College of General Practitioners (RCGP), Department of Health and the Mental Health Foundation funded a Senior Mental Health Education GP Fellowship (A.T.) at the Institute of Psychiatry (now Institute of Psychiatry, Psychology and Neuroscience) at Kings College London, to establish Trailblazers with regional GP leads, beginning in the West Midlands, Yorkshire and South-West Regions. It expanded to the North-East, East Midlands, East Anglia, South London and South-East regions, and ran for 10 years. Regional evaluations demonstrated enhanced interprofessional collaboration. Paired delegates from psychiatry and general practice co-designed services in three residential meetings each year, with multi-professional tutor and peer support. The scheme spread throughout New Zealand and New Hampshire in the USA.

Early in the new century, the National Institute for Health and Care Excellence developed guidelines for depression and anxiety disorders recommending cognitive–behavioural therapy (CBT) delivered by clinical psychologists. An unplanned consequence was widespread de-commissioning of practice counsellors, psychotherapists and counselling psychologists by primary care trusts (PCTs), in favour of clinical psychologists. PCTs often adopted a narrow definition of CBT and were reluctant to re-badge existing staff.

An economic case was made that widespread provision of CBT by a new primary care psychology service in parallel to general practice would reduce unemployment and increase tax revenue. More than a billion pounds over several years has been invested in the Improving Access to Psychological Therapies (IAPT) programme,^7^ and industrial numbers of patients have received CBT with a focus on return to work and a clinically meaningful reduction in questionnaire scores for anxiety and depression.

However, many patients with depression and anxiety referred by GPs have complex comorbid disorders (i.e. post-traumatic stress disorder, personality disorder, alcohol and substance misuse, etc.) and are not eligible for referral to IAPT. The policy paper ʻNeglected Majority'^8^ described the need for a much wider approach, confirmed in a study of patients referred to a London IAPT,^9^ in which many participants had suffered childhood abuse or neglect. There is a clear, currently unmet need for better psychiatric and psychotherapeutic care for such patients. Since April 2021, NHS England and NHS Improvement^1^ have been funding better multi-agency collaboration through the 42 English integrated care systems. Community-based mental health services will be transformed, co-designed and operated by community mental health services, primary care networks and corresponding clinical commissioning groups, third-sector providers, social care providers in local authorities, patients and carers. All of these agencies will now need to collaborate and learn from the successes of the 12 pilot schemes to date. This builds on the achievements of Trailblazers, with scope for improvement as more agencies are involved, and there is the benefit of the new Integrated Care Systems to provide community-based pathways of holistic mental healthcare.

The Royal College of Psychiatrists and the Royal College of General Practitioners have collaborated on campaigns and task groups in the past, and so can undoubtedly play a role in this new multi-agency endeavour.

## About the authors

**André Tylee,** MD, FRCGP, MRCPsych (Hon), FRCPsych, is an Emeritus Professor of Primary Care Mental Health in the Health Services and Population Research Department, Institute of Psychiatry, Psychology and Neuroscience, King's College London and the NIHR South London ARC, UK. **Alan Cohen,** FRCGP, is a retired GP and Board Member, Oxfordshire Mind, Oxford, UK. **Lydia Thurston,** MRCPsych, is a Specialty Doctor, Community Learning Disability Team, South Oxfordshire, Oxford Health NHS Foundation Trust, UK.
